# Progressive Multifocal Leukoencephalopathy in a Multiple Sclerosis Patient Diagnosed after Switching from Natalizumab to Fingolimod

**DOI:** 10.1155/2016/5876798

**Published:** 2016-11-22

**Authors:** Tim Sinnecker, Jalal Othman, Marc Kühl, Imke Metz, Thoralf Niendorf, Annett Kunkel, Friedemann Paul, Jens Wuerfel, Juergen Faiss

**Affiliations:** ^1^Department of Neurology, Asklepios Fachklinikum Teupitz, Teupitz, Germany; ^2^NeuroCure Clinical Research Center, Charité-Universitätsmedizin Berlin, Berlin, Germany; ^3^Department of Neurology, Universitätsspital Basel, Basel, Switzerland; ^4^Department of Neuropathology, Universitätsmedizin Göttingen, Göttingen, Germany; ^5^Berlin Ultrahigh Field Facility, Max Delbrück Center for Molecular Medicine, Berlin, Germany; ^6^Experimental and Clinical Research Center, Charité-Universitätsmedizin Berlin and Max Delbrück Center for Molecular Medicine, Berlin, Germany; ^7^Clinical and Experimental Multiple Sclerosis Research Center, Charité-Universitätsmedizin Berlin, Berlin, Germany; ^8^Department of Neurology, Charité-Universitätsmedizin Berlin, Berlin, Germany; ^9^Medical Imaging Analysis Center AG, Basel, Switzerland

## Abstract

*Background*. Natalizumab- (NTZ-) associated progressive multifocal leukoencephalopathy (PML) is a severe and often disabling infectious central nervous system disease that can become evident in multiple sclerosis (MS) patients after NTZ discontinuation. Recently, novel diagnostic biomarkers for the assessment of PML risk in NTZ treated MS patients such as the anti-JC virus antibody index have been reported, and the clinical relevance of milky-way lesions detectable by MRI has been discussed.* Case Presentation and Conclusion*. We report a MS patient in whom PML was highly suspected solely based on MRI findings after switching from NTZ to fingolimod despite repeatedly negative (ultrasensitive) polymerase chain reaction (PCR) testing for JC virus DNA in cerebrospinal fluid. The PML diagnosis was histopathologically confirmed by brain biopsy. The occurrence of an immune reconstitution inflammatory syndrome (IRIS) during fingolimod therapy, elevated measures of JCV antibody indices, and the relevance of milky-way-like lesions detectable by (7 T) MRI are discussed.

## 1. Introduction

Progressive multifocal leukoencephalopathy (PML) is an opportunistic infection of the central nervous system (CNS) caused by JC polyomavirus (JCV) targeting oligodendrocytes and astrocytes and leading to oligodendrocyte death [1]. Symptoms are greatly variable, depending on the localisation of the infection in the brain [2]. Clinically, patients present with behavioural abnormalities, cognitive impairment, focal neurological deficits, and/or epileptic seizures. The course of the disease is often fatal or rendering the patient severely disabled [2].

PML is observed in patients with a marked immunosuppression, for instance, due to an infection with HIV or as a result of an immunosuppressive therapy after organ transplantation. It may also occur in multiple sclerosis (MS) patients treated with natalizumab (NTZ). NTZ is a monoclonal antibody directed against *α*4-integrin that hinders the transmigration of white blood cells through the blood vessel wall into the CNS. Risk factors of NTZ-associated PML are duration of therapy with NTZ (with a marked increase in risk after two years), use of immunosuppressants before initiation of NTZ therapy, and a positive anti-JC virus antibody status [3–7].

After clinical suspicion of PML, diagnosis is established by magnetic resonance imaging (MRI) findings and PCR detection of JCV DNA in the cerebrospinal fluid (CSF) [8]. In rare cases, a brain biopsy has to be performed to diagnose PML [8].

Apart from reestablishing a competent immune response, there is no PML-specific therapy with proven efficacy [9]. In MS patients with NTZ-associated PML, plasma exchange (PLEX) or immunoadsorption (IA) is performed to accelerate NTZ clearance [10]. However, immune reconstitution inflammatory syndrome (IRIS), a condition characterized by an overwhelming inflammatory response during immune reconstitution, can develop or deteriorate during PLEX leading to clinical worsening [11].

In vitro studies postulate an infection via the serotonin receptor 5HT2a [12]. Hence, serotonin reuptake inhibitors like mirtazapine are frequently prescribed. However, along with other experimental therapeutic strategies including mefloquine or amantadine, clinical confirmation is still missing [13].

Here, we report an MS case in which PML-IRIS was diagnosed after switching from NTZ to fingolimod. Brain biopsy and advanced neuroimaging findings including ultrahigh field MRI at 7 Tesla (T) are presented.

## 2. Case Presentation

A 48-year-old woman with relapsing-remitting MS (RRMS) was switched after 6 months of treatment with interferon-1b to NTZ in May 2008 due to ongoing clinical and paraclinical disease activity including multiple Gadolinium enhancing brain lesions detected with MRI.

At that point, the Expanded Disability Status Scale Score (EDSS) was 5.5.

We did not observe any evidence of clinical or MRI disease activity during NTZ treatment, and the EDSS subsequently decreased to 2.5.

**Figure 1:**
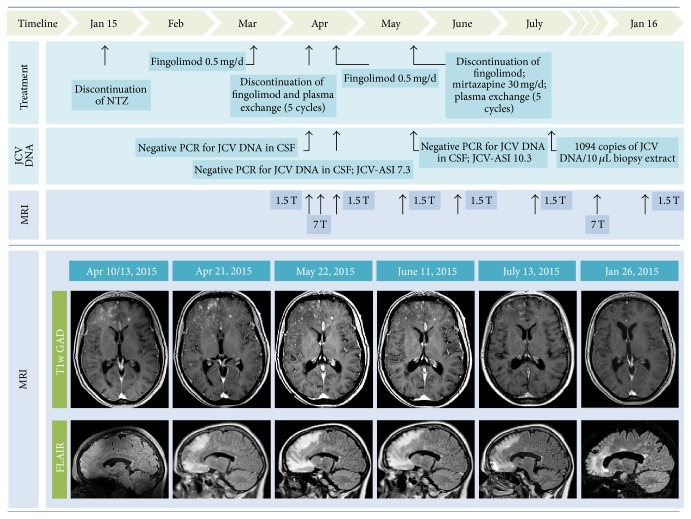
Overview. Survey of treatment decisions and laboratory and MRI findings of the patient under discussion. In detail, 1.5 T T1 weighted Gadolinium enhanced (T1W GAD) and 1.5 T fluid attenuated inversion recovery (FLAIR) images are presented.

Figure 1 chronologically summarizes all paraclinical findings including MRI results and treatment decisions.

In January 2015, NTZ was discontinued after a total of 86 infusions on the background of seroconversion to positive JCV serum antibodies (STRATIFY, Unilabs, Geneva, Switzerland), indicating an increased PML risk. Anti-JCV antibody index was not available at that time.

After a wash-out period of 2 months, fingolimod was started on the 18th of March 2015. Previous brain MRI (February 2015) did not show any signs of PML.

**Figure 2:**
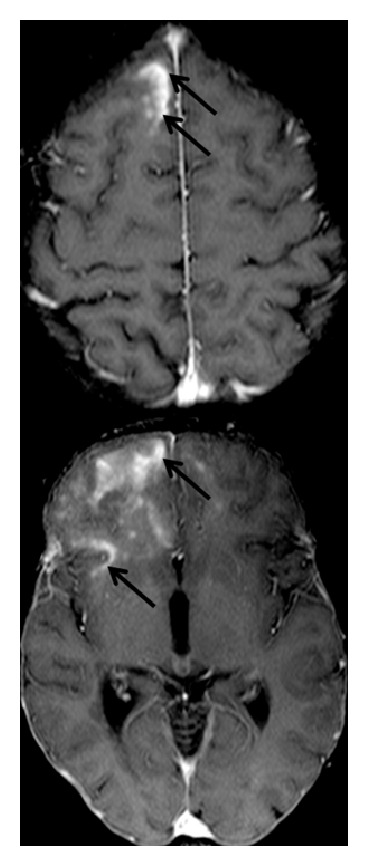
Signs of IRIS at the time of PML diagnosis. 1.5 T T1w Gadolinium enhanced MR images are presented. Extensive Gadolinium enhancement suggestive of IRIS (black arrows) was observed at the edge of confluent PML lesions.

**Figure 3:**
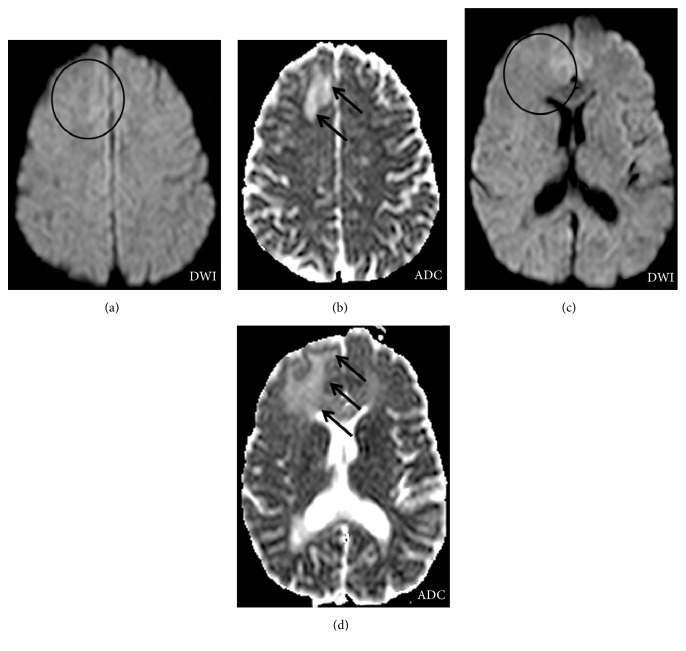
No signs of abnormal diffusion. Diffusion weighted MRI at 1.5 T ((a) and (c)) did reveal neither signs of central hyperdiffusibility (circles) nor signs of restricted diffusion (circles) at the edge of PML lesions (black arrows, (b) and (d)). Both of which were reported to be characteristic for PML lesions.

Three weeks later (10th April 2015), routine brain MRI at 1.5 T revealed PML-suspicious bifrontal confluent lesions with (sub)cortical involvement. Moreover, multiple milky-way-like Gadolinium enhancing and T2 weighted (T2w) hyperintense punctate lesions were detected by MRI in these areas (Figure 1). In addition, perilesional contrast enhancement around confluent PML-suspicious lesions suggestive of IRIS was detectable (Figure 2). Diffusion weighted MRI did not show intralesional hyperdiffusivity nor signs of restricted diffusion at the edge of the lesions (Figure 3); both of which are considered to be typical of PML [14].

We did not observe any signs of clinical worsening, the polymerase chain reaction (PCR) testing for JCV DNA in CSF* (Institute for Virology, Heinrich Heine University, Düsseldorf, Germany)* was negative, and the lymphocyte count was only slightly decreased (0.91 G/L, reference range 1–4 G/L).

**Figure 4:**
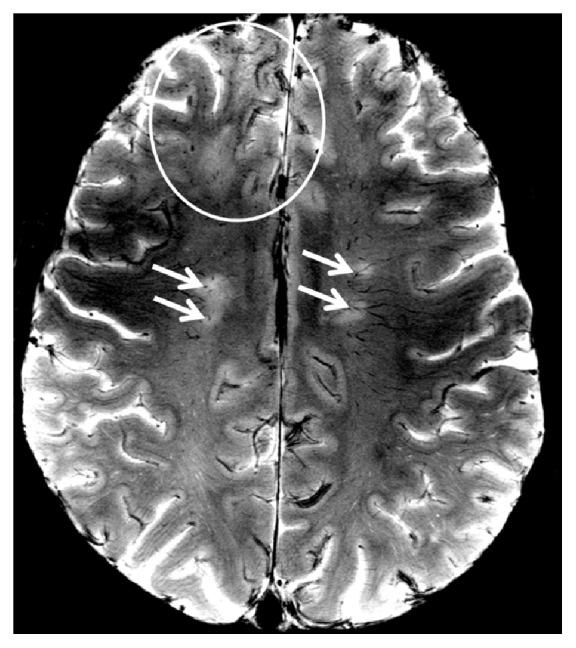
7 T T2^**∗**^ weighted imaging in PML. A 7 T T2^**∗**^ weighted (T2^**∗**^w) image with a spatial resolution of (0.2 × 0.2) mm is shown. Please note the difference in lesion morphology between periventricular oval MS lesions that are centered on a small venous vessel (arrows) and confluent PML lesions (circle) that also involve U-fibers and subcortical areas.

**Figure 5:**
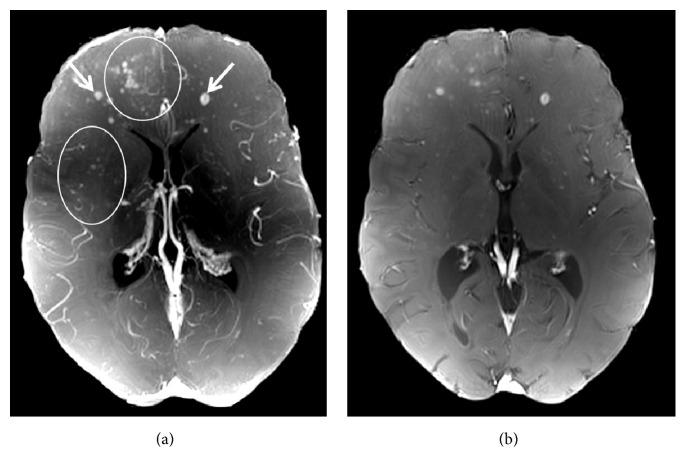
Patterns of Gadolinium enhancement on 7 T VIBE images. A maximum intensity projection map of a 7 T T1 weighted Gadolinium enhanced volumetric interpolated brain examination ((a), VIBE) and an exemplary VIBE image (b) are displayed. PML-suspicious punctate Gadolinium enhancing lesions are clearly visible (circles). Ring-enhancing lesions (e.g., arrows) suggestive of MS lesions are delineated.

Fingolimod was immediately discontinued, and the patient underwent five cycles of plasma exchange. Ultrahigh field MRI at 7 Tesla was performed five days after discontinuation of fingolimod confirming initial 1.5 T MRI findings by detailing confluent PML-suspicious lesions with (sub)cortical involvement (Figure 4) and by delineating numerous punctate Gadolinium enhancing lesions (Figure 5, circles) on top of MS-suspicious ring-enhancing lesions (Figure 5, white arrows).

1.5 T MRI performed immediately after PLEX did not show any signs of PML progression (Figure 1), and PCR did again not reveal JCV DNA in CSF. Thus, fingolimod was reinitiated on 22th of April 2015 to prevent possible rebound effects after discontinuation of NTZ, and monthly MRIs were performed.

One month later (22th of May 2015) a control MRI at 1.5 T showed slightly enlarging FLAIR hyperintense lesions (Figure 1). Clinically, we observed a latent right-sided brachiofacial paresis and a slightly increased irritability reported by her daughter at that time; EDSS 3.0. PCR testing for JCV DNA in CSF was repeatedly negative, but JCV antibody index (JCV-ASI) was markedly increased (10.3). Retrospectively, JCV-ASI was already elevated at the time of the second CSF analysis (JCV-ASI 7.3).

As a consequence, fingolimod was again discontinued, mirtazapine 30 mg/d orally was started, and another cycle of plasma exchange was carried out. Neuropsychological examinations and electroencephalography (EEG) did not reveal any changes.

**Figure 6:**
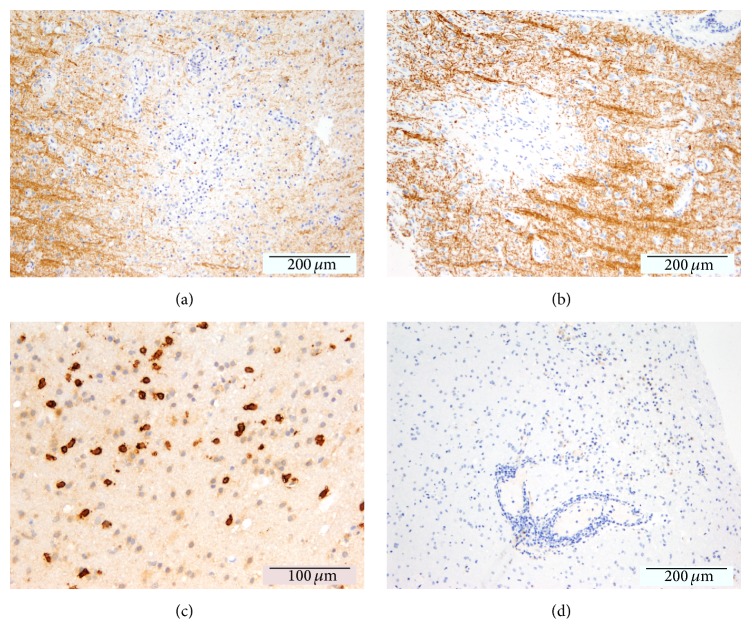
Neuropathological findings. Histology revealed areas of focal demyelination as indicated by a loss of myelin basic protein (a) and proteolipid protein (b). Despite the presence of prominent CD8 dominated inflammatory infiltrates (c), SV40-positive cells (JCV-infected cells, (d)) could not be detected.

On 24th of July 2015, a stereotactic biopsy was carried out since an ultrasensitive PCR of JCV DNA* (Laboratory of Molecular Medicine and Neuroscience, National Institute of Health, Bethesda, USA)* repetitively failed to detect JCV DNA in CSF. The biopsy showed demyelinating lesions with a prominent CD8 dominated inflammatory infiltrate with numerous plasma cells (Figure 6). Although neuropathological findings were highly suggestive of IRIS in the context of PML, SV40-positive cells (JCV-infected cells) could not be detected* (Institute of Neuropathology, University of Göttingen, Germany)*. JCV multiplex quantitative real-time PCR assay (JC Multiplex qPCR) of paraffin embedded brain tissue was initiated and revealed 1094 viral copies per 10 *μ*L extract, consistent with a variant most commonly associated with PML* (Laboratory of Molecular Medicine and Neuroscience, National Institute of Health, Bethesda, USA)* [14], finally proving the PML diagnosis.

Mirtazapine was continued and glatiramer acetate treatment initiated. The patient remained clinically stable, and MRI (26th of January 2016) showed decreasing PML lesions without any signs of Gadolinium enhancement (Figure 1, EDSS 3.0).

## 3. Discussion

We report a case of subclinical simultaneous PML-IRIS that was diagnosed after switching from NTZ to fingolimod. Initially, PML was suspected exclusively on the basis of MRI findings despite repeatedly negative (ultrasensitive) PCR testing for JCV DNA in CSF. The diagnosis was further complicated by the absence of PML-characteristic changes in diffusivity as investigated by diffusion weighted MRI. Finally, PML was confirmed via brain biopsy.

Along with other reports in the literature [15, 16], this case thus underlines the need of additional sensitive biomarkers for an earlier diagnosis of PML. In fact, PCR testing for JCV DNA in CSF is limited in sensitivity even when using ultrasensitive PCR assays that can detect up to 10 copies of JCV DNA per milliliter CSF [8, 16]. Notwithstanding these efforts, such highly sensitive assays are not broadly available, and the clinical relevance of very low measures of JCV DNA copies is still under discussion [17].

Recently, the JCV antibody index was introduced as a novel biomarker that potentially can help to better distinguish between NTZ-associated PML and non-PML MS patients [4, 16]. Indeed, the JCV antibody index was markedly increased in our case and continued to rise during PML expansion. Other PML cases of elevated JCV antibody indices despite repeatedly negative PCR testing for JCV DNA in CSF have been reported [15, 16].

Furthermore, the presented case also highlights the importance of a stringent clinical and paraclinical follow-up of MS patients before and after discontinuing NTZ since PML(-IRIS) was previously described after NTZ discontinuation [18] and while switching from NTZ to another immunomodulatory therapy. As reported previously, IRIS may even occur during fingolimod-associated lymphopenia [19, 20]. Indeed, marginally decreased blood lymphocyte counts and signs of IRIS were detectable at the time of first PML-suspicious MRI lesions in our case.

In addition to this extensive laboratory and clinical workup, we performed highly resolving ultrahigh field MRI at 7 T. In general, 7 T MRI benefits from an increased signal-to-noise ratio, a high spatial resolution, and enhanced susceptibility effects. Thus, 7 T MRI has improved the detection and morphological characterization of neuroinflammatory brain lesions [21–23]. Most importantly, a small venous vessel is often detectable within the center of MS lesions by using gradient echo MR techniques at 7 T [24–28], facilitating the distinction to other CNS diseases such as neuromyelitis optica [29, 30] and Susac syndrome [31].

Recently, 7 T MRI revealed contrast-enhancing milky-way-like lesions that expanded into more typical PML lesions over time in a single case of simultaneous PML, IRIS, and an ongoing MS disease activity [32]. In contrast to MS lesions, a small central vessel was not commonly detectable within these lesions [32].

In general, the mechanisms of contrast enhancement in NTZ-associated PML are not fully understood. Contrast enhancement is a correlate of blood-brain-barrier (BBB) breakdown [11, 13, 33–35]. However, JCV-infected lymphocytes may also cross the intact BBB to infect oligodendrocytes [36, 37]. In other words, BBB breakdown is not a prerequisite of PML development. In HIV, indeed, PML is frequently characterized by little or no inflammatory signs and absence of BBB breakdown [38]. Thus, patchy areas of peripheral contrast enhancement at the edge of HIV-PML lesions are commonly considered as a sign of IRIS but not a PML imaging feature [38, 39]. Following this assumption, recent PML studies have interpreted any kind of contrast enhancement in or around PML lesions as a sign of IRIS [11]. However, it is not known whether this also holds true for NTZ-associated PML, where the immune response is present and thus different compared to HIV. In a recent report, no histopathological features of IRIS were present in a bioptical probe of NTZ-associated PML, despite perilesional contrast enhancement on MRI. The authors concluded that, up to date, IRIS remains a histopathological diagnosis [40]. In our case, histopathology revealed prominent CD8 dominated inflammatory infiltrates with numerous plasma cells highly suggestive of IRIS, although clinical worsening, that usually accompanies IRIS, was absent.

In addition to patchy contrast enhancement at the edges of PML lesions, punctate contrast-enhancing lesions have been described [35, 41–43]. The clinical relevance of such small punctate lesion is, however, still a matter of discussion: On the one hand, milky-way-like punctate lesions were associated with an overwhelming immunoreaction, namely, IRIS, against JCV [43]. Methylprednisolone pulse therapy would be beneficial in such a situation. On the other hand, it was hypothesized that these lesions represent areas of active JCV replication that is probably adequately recognized by the immune system [41]. In such a scenario, glucocorticoid induced immunosuppression might be harmful. In alignment with this hypothesis, we have previously described clinical worsening and increasing JCV DNA copies in CSF in a NTZ-PML case with punctate lesions during methylprednisolone pulse therapy [32].

Interestingly, there are some differences in the clinical presentation and MRI finding between the “current” PML case presented here and the previous one [32]. In detail, we observed fewer milky-way-like lesions and the expansion of confluent lesions over time was more limited in the “current” case. Of note, the “current” patient only received plasma exchange, and she was not treated with methylprednisolone. Which of all these factors has primarily influenced the overall better clinical outcome of the presented patient remains unknown, but it emphasizes the need of systematic (ultra)high field MRI studies to address these questions.

## References

[1] Richardson-Burns S. M., Kleinschmidt-DeMasters B. K., DeBiasi R. L., Tyler K. L. (2002). Progressive multifocal leukoencephalopathy and apoptosis of infected oligodendrocytes in the central nervous system of patients with and without AIDS. *Archives of Neurology*.

[2] Vermersch P., Kappos L., Gold R. (2011). Clinical outcomes of natalizumab-associated progressive multifocal leukoencephalopathy. *Neurology*.

[3] Bloomgren G., Richman S., Hotermans C. (2012). Risk of natalizumab-associated progressive multifocal leukoencephalopathy. *The New England Journal of Medicine*.

[4] Schwab N., Schneider-Hohendorf T., Pignolet B. (2016). Therapy with natalizumab is associated with high JCV seroconversion and rising JCV index values. *Neurology - Neuroimmunology Neuroinflammation*.

[5] Meira M., Sievers C., Hoffmann F. (2016). Natalizumab-induced POU2AF1/Spi-B upregulation. *Neurology—Neuroimmunology Neuroinflammation*.

[6] Major E. O., Nath A. (2016). A link between long-term natalizumab dosing in MS and PML: putting the puzzle together. *Neurology-Neuroimmunology Neuroinflammation*.

[7] Javed A., Reder A. T. (2016). Rising JCV-Ab index during natalizumab therapy for MS: inauspicious for a highly efficacious drug. *Neurology: Neuroimmunology & Neuroinflammation*.

[8] Berger J. R., Aksamit A. J., Clifford D. B. (2013). PML diagnostic criteria: consensus statement from the AAN neuroinfectious disease section. *Neurology*.

[9] Du Pasquier R. A., Kuroda M. J., Zheng Y., Jean-Jacques J., Letvin N. L., Koralnik I. J. (2004). A prospective study demonstrates an association between JC virus-specific cytotoxic T lymphocytes and the early control of progressive multifocal leukoencephalopathy. *Brain: A Journal of Neurology*.

[10] Khatri B. O., Man S., Giovannoni G. (2009). Effect of plasma exchange in accelerating natalizumab clearance and restoring leukocyte function. *Neurology*.

[11] Tan I. L., McArthur J. C., Clifford D. B., Major E. O., Nath A. (2011). Immune reconstitution inflammatory syndrome in natalizumab-associated PML. *Neurology*.

[12] Elphick G. F., Querbes W., Jordan J. A. (2004). The human polyomavirus, JCV, uses serotonin receptors to infect cells. *Science*.

[13] Clifford D. B., DeLuca A., Simpson D. M., Arendt G., Giovannoni G., Nath A. (2010). Natalizumab-associated progressive multifocal leukoencephalopathy in patients with multiple sclerosis: lessons from 28 cases. *The Lancet Neurology*.

[14] Hodel J., Outteryck O., Dubron C. (2016). Asymptomatic progressive multifocal leukoencephalopathy associated with natalizumab: diagnostic precision with MR imaging. *Radiology*.

[15] Kuhle J., Gosert R., Bühler R. (2011). Management and outcome of CSF-JC virus PCR-negative PML in a natalizumabtreated patient with MS. *Neurology*.

[16] Warnke C., von Geldern G., Markwerth P. (2014). Cerebrospinal fluid JC virus antibody index for diagnosis of natalizumab-associated progressive multifocal leukoencephalopathy. *Annals of Neurology*.

[17] Iacobaeus E., Ryschkewitsch C., Gravell M. (2009). Analysis of cerebrospinal fluid and cerebrospinal fluid cells from patients with multiple sclerosis for detection of JC virus DNA. *Multiple Sclerosis*.

[18] Gheuens S., Smith D. R., Wang X., Alsop D. C., Lenkinski R. E., Koralnik I. J. (2012). Simultaneous PML-IRIS after discontinuation of natalizumab in a patient with MS. *Neurology*.

[19] Killestein J., Vennegoor A., van Golde A. E. L., Bourez R. L. J. H., Wijlens M. L. B., Wattjes M. P. (2014). PML-IRIS during fingolimod diagnosed after natalizumab discontinuation. *Case Reports in Neurological Medicine*.

[20] Calic Z., Cappelen-Smith C., Hodgkinson S. J., McDougall A., Cuganesan R., Brew B. J. (2015). Treatment of progressive multifocal leukoencephalopathy-immune reconstitution inflammatory syndrome with intravenous immunoglobulin in a patient with multiple sclerosis treated with fingolimod after discontinuation of natalizumab. *Journal of Clinical Neuroscience*.

[21] Sinnecker T., Kuchling J., Dusek P. (2015). Ultrahigh field MRI in clinical neuroimmunology: a potential contribution to improved diagnostics and personalised disease management. *EPMA Journal*.

[22] Sinnecker T., Mittelstaedt P., Dörr J. (2012). Multiple sclerosis lesions and irreversible brain tissue damage: a comparative ultrahigh-field strength magnetic resonance imaging study. *Archives of Neurology*.

[23] Kuchling J., Ramien C., Bozin I. (2014). Identical lesion morphology in primary progressive and relapsing-remitting MS -an ultrahigh field MRI study. *Multiple Sclerosis Journal*.

[24] Bozin I., Ge Y., Kuchling J. (2015). Magnetic resonance phase alterations in multiple sclerosis patients with short and long disease duration. *PLoS ONE*.

[25] Müller K., Kuchling J., Dörr J. (2014). Detailing intra-lesional venous lumen shrinking in multiple sclerosis investigated by sflair mri at 7-t. *Journal of Neurology*.

[26] Sinnecker T., Bozin I., Dörr J. (2013). Periventricular venous density in multiple sclerosis is inversely associated with T2 lesion count: a 7 Tesla MRI Study. *Multiple Sclerosis Journal*.

[27] Blaabjerg M., Ruprecht K., Sinnecker T. (2016). Widespread inflammation in CLIPPERS syndrome indicated by autopsy and ultra-high-field 7T MRI. *Neurology-Neuroimmunology Neuroinflammation*.

[28] Sinnecker T., Schumacher S., Mueller K. (2016). MRI phase changes in multiple sclerosis vs neuromyelitis optica lesions at 7T. *Neurology Neuroimmunology Neuroinflammation*.

[29] Sinnecker T., Dörr J., Pfueller C. F. (2012). Distinct lesion morphology at 7-T MRI differentiates neuromyelitis optica from multiple sclerosis. *Neurology*.

[30] Kister I., Herbert J., Zhou Y., Ge Y. (2013). Ultrahigh-field MR (7 T) imaging of brain lesions in neuromyelitis optica. *Multiple Sclerosis International*.

[31] Wuerfel J., Sinnecker T., Ringelstein E. B. (2012). Lesion morphology at 7 Tesla MRI differentiates Susac syndrome from multiple sclerosis. *Multiple Sclerosis Journal*.

[32] Sinnecker T., Othman J., Kühl M. (2015). 7T MRI in natalizumab-associated PML and ongoing MS disease activity: a case study. *Neurology: Neuroimmunology & Neuroinflammation*.

[33] Wattjes M. P., Wijburg M. T., Vennegoor A. (2016). MRI characteristics of early PML-IRIS after natalizumab treatment in patients with MS. *Journal of Neurology, Neurosurgery & Psychiatry*.

[34] Wattjes M. P., Richert N. D., Killestein J. (2013). The chameleon of neuroinflammation: magnetic resonance imaging characteristics of natalizumab-associated progressive multifocal leukoencephalopathy. *Multiple Sclerosis Journal*.

[35] Hodel J., Darchis C., Outteryck O. (2016). Punctate pattern: a promising imaging marker for the diagnosis of natalizumab-associated PML. *Neurology*.

[36] Sabath B. F., Major E. O. (2002). Traffic of JC virus from sites of initial infection to the brain: the path to progressive multifocal leukoencephalopathy. *Journal of Infectious Diseases*.

[37] Saribaş A. S., Özdemir A., Lam C., Safak M. (2010). JC virus-induced progressive multifocal leukoencephalopathy. *Future Virology*.

[38] Bag A. K., Curé J. K., Chapman P. R., Roberson G. H., Shah R. (2010). JC virus infection of the brain. *American Journal of Neuroradiology*.

[39] Tan K., Roda R., Ostrow L., McArthur J., Nath A. (2009). PML-IRIS in patients with HIV infection: clinical manifestations and treatment with steroids. *Neurology*.

[40] Metz I., Radue E.-W., Oterino A. (2012). Pathology of immune reconstitution inflammatory syndrome in multiple sclerosis with natalizumab-associated progressive multifocal leukoencephalopathy. *Acta Neuropathologica*.

[41] Wattjes M. P., Verhoeff L., Zentjens W. (2013). Punctate lesion pattern suggestive of perivascular inflammation in acute natalizumab-associated progressive multifocal leukoencephalopathy: productive JC virus infection or preclinical PML-IRIS manifestation?. *Journal of Neurology, Neurosurgery and Psychiatry*.

[42] Wattjes M. P., Vennegoor A., Steenwijk M. D. (2015). MRI pattern in asymptomatic natalizumab-associated PML. *Journal of Neurology, Neurosurgery & Psychiatry*.

[43] Yousry T. A., Pelletier D., Cadavid D. (2012). Magnetic resonance imaging pattern in natalizumab-associated progressive multifocal leukoencephalopathy. *Annals of Neurology*.

